# Shaping and Designing Health Communication Messages Around Culture in the Kingdom of Saudi Arabia

**DOI:** 10.7759/cureus.63874

**Published:** 2024-07-05

**Authors:** Najla Alhraiwil, Mohammed A Ba Oshra, Mohammed S Aldossary

**Affiliations:** 1 Public Health, Saudi Arabia Ministry of Health, Riyadh, SAU; 2 Communication, Saudi Arabia Ministry of Health, Riyadh, SAU; 3 Research and Studies, Saudi Arabia Ministry of Health, Riyadh, SAU

**Keywords:** vaccination, social media, healthy lifestyles, health campaigns, saudi arabia

## Abstract

The Kingdom of Saudi Arabia launched several health campaigns tailored to the Saudis’ culture over the past few years. These campaigns drew attention toward shifting to and maintaining healthy lifestyles and health-disorder management, particularly diabetes. Almost all the campaigns achieved success stories. These success stories were manifested by fruitful outcomes such as increasing vaccination rates and receiving awards (i.e., the “Marketing Pioneers Award”). This paper presents the development strategy and communicates the most recently culturally adapted health campaigns implemented by the Kingdom of Saudi Arabia Ministry of Health.

## Introduction

Health programs aim to increase education about health issues and managing health problems or maintaining specific health status. However, a mismatch between health program objectives and real experiences by people could lead to unsuccessful outcomes such as an increase in chronic diseases, far from reaching vaccination targets, and failure to eradicate health risk factors [1,2]. From an anthropological point of view, different milieus of individuals within and across countries and cultures shape their perspectives and health-related choices [2]. Diverse populations’ beliefs and perspectives are the products of socio-political and cultural adaptations throughout the life course of individuals and groups.

Recently, Tan et al. described “The Revised Theoretical Framework” of the health belief model. They incorporated six cultural appropriateness strategies into a health message's linguistic and content aspects [3]. These cultural appropriateness strategies include cultural identity (the core of the health intervention), perceptual features (culturally appealing audiovisuals), constituent-involving (the involvement of the audience), and the socioeconomic context-adaptive (wider determinants of health that could be protective such as equal access to healthcare facilities or preventive such as poor healthcare transportation system). Intrigued by the revised theoretical framework of the health belief model, the Saudi Ministry of Health (MoH) has identified the necessity of cultural adaptations in its own country [4-6]. It has undertaken several public health and health communication initiatives while incorporating cultural aspects unique to its renowned Arabic, Islamic, and Gulf traditions and societies [4-6]. Success stories in this regard have been reported [4-6]. The Saudi MoH attended keenly to integrate Saudi culture into health awareness campaigns and marketing strategies [4-6]. In this health communication paper, we aim to convey the health awareness campaigns conducted in the Kingdom of Saudi Arabia (KSA) to set the stage for implementing more successful health campaigns tailored to the country’s culture and society.

## Materials and methods

Mass media health campaign process

The awareness, marketing, and production departments worked collectively and diligently on producing the mass media health campaigns. The awareness department team involved health education, promotion, and public health specialists to lead the projects. They also provided copywriters, graphic designers, and the medical committee. The marketing department team, alongside provided marketing specialists and the creative studio equipped with creative content creators. Last comes the production department team to seal the process by writing and scripting as well as film producing. All team members worked in harmony and swiftly owing to their extensive experience and motivation toward the health campaigns’ production and dissemination.

The initial step of the process commences whenever the MoH specifies the need for a specific health campaign. At the outset, the director of the awareness department assigns a campaign manager to procure and supervise the health awareness campaign. The manager will review the scope, the goal, and the targeted population of the campaign. This is followed by directing the team experts to start designing the scientific strategies for implementation. In addition, the manager will involve the marketing department to develop and expand the marketing plan. On the other hand, the creative studio team builds an integrated file for the campaign and develops the campaign style and the idea of the promotional products. The creative writers then start writing the appropriate content, while the designers put this content into a graphic design. Lastly, the production department produces a video based on the idea, content, and design.

The awareness campaign manager reviews and approves the content, designs, and videos from the previous entries in addition to the publishing plan. Approval of the final products is sought from the medical committee, the general director of the marketing and awareness department, and the quality team.

Overview of the KSA health campaigns (2016-2022)

The Camel Auction Festival (Hail Region) (2022)

**Figure 1:**
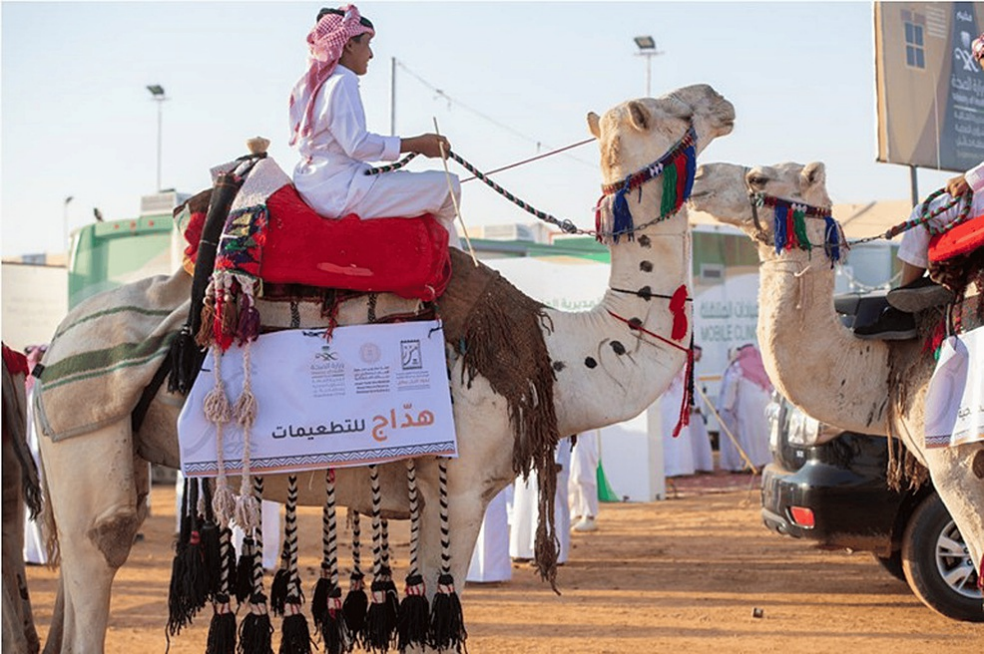
The Camel Auction Festival (Hail region) (2022) Source: SBQ Online Newspaper (https://sabq.org/saudia/jl2x485m2p)

Camels in KSA symbolize history and tradition for Saudis and have been the Saudi’s companions for thousands of years [7]. Dr. Abdullah Alsharekh from the Department of Archaeology at King Saud University mentioned that camels are not only history but continue to have a significant role in modern society [7]. Its role is manifested through breeding and as a source of food and traditional materials. In addition, Dr. Alsharekh added, “Camels are mentioned in the Holy Qur’an,” and from here, the religious value of camels emerges [7]. In October 2022, the Imam Turki bin Abdullah Royal Reserve Development Authority’s Festival for Camel Auction took place in the Hail region of KSA. Health tents were introduced during this festival and targeted camel owners and visitors. Through these tents, several health educational messages and services were provided. Interestingly, the camels were used as a cultural carrier of these health activities as a marketing advertising strategy tailored to the camel’s tradition and cultural meaning (Figure 1).

COVID-19 Fourth Vaccination Campaign (2021)

**Figure 2:**
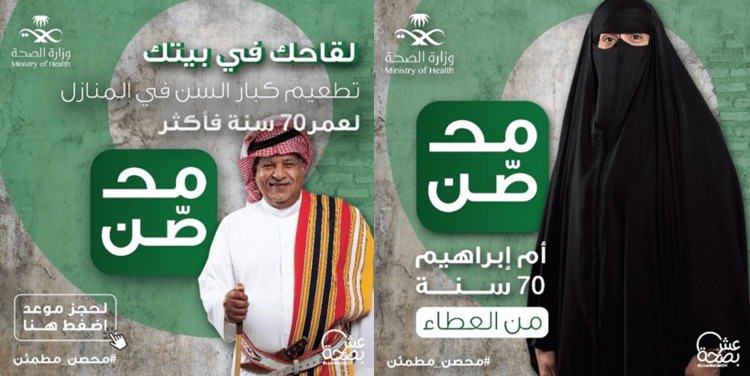
COVID-19 fourth vaccination campaign (2021) Source: Ministry of Health Twitter [8]

The Saudi cultural costumes are characterized by the traditional clothing of “Abaya” and “Shayla” for women and “Thawb” for men. Awareness of how important this tradition is to the Saudi nation is a step toward acknowledging their culture and standards. During the fourth COVID-19 vaccination campaign, the MoH integrated this tradition into visual advertisements, be it photographs or videos. The fourth campaign was dedicated to the elderly and reminded of the second booster dose, after which the first, second, and third campaigns were inaugurated with similar profiles and unique launches. The highly interesting COVID-19 campaigns’ ads and proceedings can be accessed through their Twitter pages [8] (Figure 2).

For You and Your Family (2021)

This health awareness campaign was launched in 2021 and tackled misconceptions about the influenza vaccine. It featured a cartoon-like figure of a doctor with a Saudi look in a video through a question-and-answer mode.

"Lose Your Habits" (2018)

**Figure 3:**
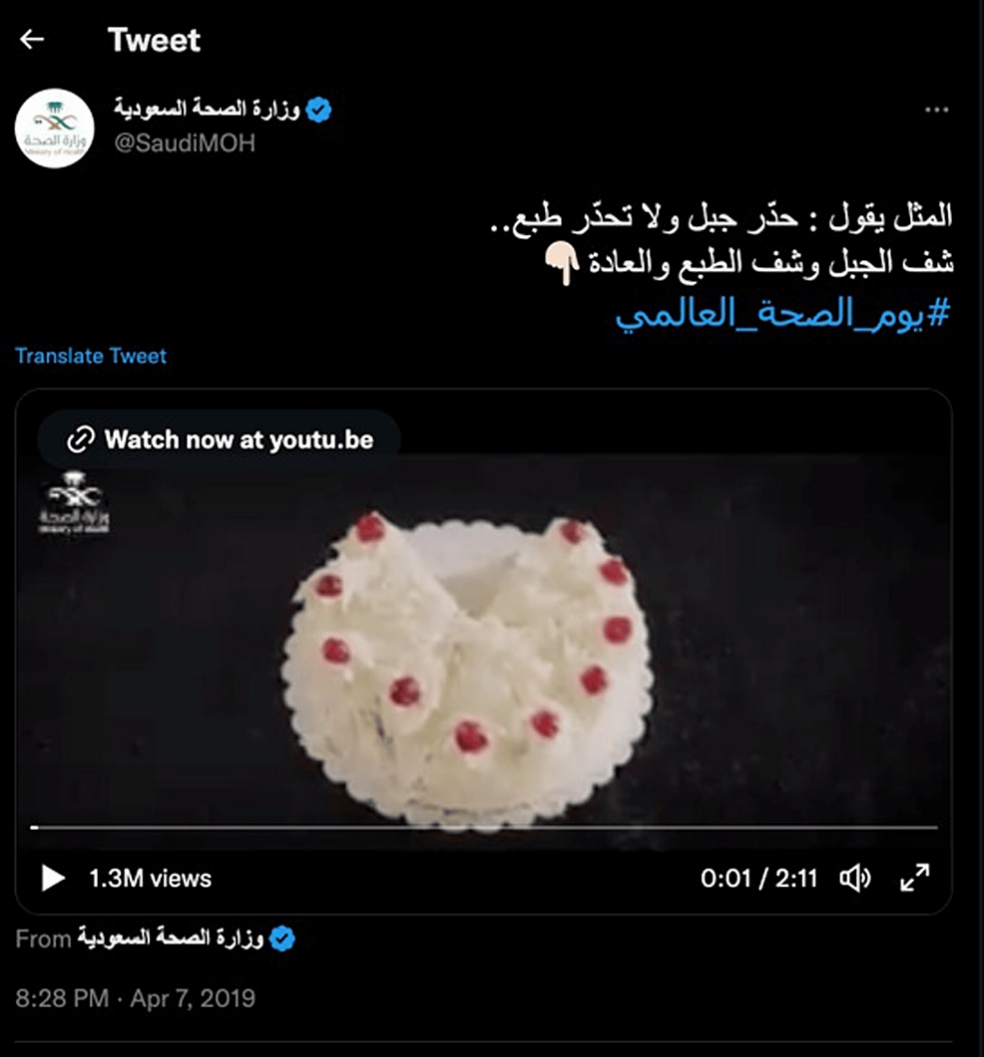
"Lose Your Habits" campaign (2018)

The “Lose Your Habits” campaign targeted individuals' habits and behaviors threatening their health. As its name implies, it advocates for getting rid of specific habits adopted by individuals that are dangerous to their health and mainly target diabetes. These habits and behaviors, such as a sedentary lifestyle, eating many sweets, and adding too much sugar to daily beverages including tea, which is very familiar to the Saudi culture, are considered risk factors for developing diabetes or diabetes complications. Scientifically, studies have extensively researched and investigated the relationship between high sugar diet and developing diabetes/complications and other health conditions [9-13]. Efforts to minimize the drastic effects of adopting such behaviors and habits are also extensive, and awareness programs worldwide work hard toward that. However, not all campaigns target individuals by referring to their cultural norms and beliefs, especially in the elderly, whose lifestyle is usually more sedentary than other age groups [14]. To that end, the KSA MoH introduced popular cultural proverbs into the health messages so people can easily understand them and relate them to everyday activities. This campaign was set out and reached the public through several means. We want to highlight also that the music and lyrics associated with the video soundtrack are pleasing to the ear and musical using the Saudi dialect. It is very appealing to both young and elderly age groups. Twitter was one of the most popular social media platforms used in KSA for this campaign, besides being used for all the COVID-19 campaigns mentioned earlier (Figure 3).

The two previous campaigns were inaugurated in 2016 and 2017 to raise awareness about diabetes in a very nuanced approach. In both campaigns, the MoH used an innovative and creative way to deliver the health message behind protecting oneself from type 2 diabetes and its progression related to the lifestyle and diet or its complications had it been genetically acquired. The MoH used the famous Joker character in the Batman movie as the primary actor of the campaign video releases on social media and other audiovisual characters. The Joker character speaks in the local Saudi dialect and wears a local dress to increase the engagement with Saudis relevant to their cultural and linguistic look. Below are the two campaigns in chronological order.

"Hold It In Check" (Diabetes Monster) (2016)

The “Hold It in Check” campaign was launched in conjunction with World Diabetes Day in 2016 [15]. The campaign adopted a certain strategy to use more creative and effective ideas. A provocative character was created and played, in order to communicate reversal awareness messages, engage target groups, and reward outstanding performers.

"Don't Wait for Diabetes" (2017)

The campaign was launched in conjunction with World Diabetes Day in November 2017 [16]. The campaign video followed the same sequel of the “Hold it in check” campaign, yet this time it showed that the diabetes character (diabetes monster) began to feel sad due to the positive behavioral changes that occurred among the Saudi community in paying attention to the diabetes and its causes and therefore trying to live in a healthy way through exercise and consuming more healthy choices.

## Results

Impact of the campaigns

The five campaigns received high interactions from the public. In addition to this, some of the campaigns received valuable prizes. For instance, the “Lose Your Habits” campaign won the 2019 third-place Marketing Pioneers Award for the “Best Governmental Campaign," a national award affiliated with the Marketing Association - a Saudi non-profit association. In previous years, stories of success received national and international prizes won by the “Hold It in Check” and the “Don’t Wait for Diabetes” campaigns in 2017 and 2018, respectively. After the “Hold It in Check/Diabetes Monster” film was broadcast through national TV and social media, a public survey was shared online with the Saudi community to measure the reach and effectiveness of the campaign, and 80% of respondents said they were convinced of the message to start exercising and some have already started.

In addition, the film won an international award from “The Creative Floor,” the Gulf “Best Governmental Communication Campaign” award by the “International Government Communication Centre,” the international “Silver” award from the “New York Festival AME Awards,” and the national “Best Governmental Communication Campaign” award affiliated with the Marketing Association in KSA. The “Don’t Wait for Diabetes” campaign won the international “Bronze” award by EFFIE awards.

Regarding the fourth COVID-19 campaign, it achieved added success to the highly impactful previous three campaigns, where it contributed to a 10% increase in second dose uptake. Results were evaluated by comparing the number of participants who received the initial dose with those who received the second dose after the campaign. This campaign reached 15 million views through social media platforms and more than 286 thousand interactions with the campaign posts.

## Discussion

Capturing cultural differences into health communication procedures is powerful. The context culture shapes particular beliefs and life features associated with people’s perceptions of health and health-seeking behaviors. Thus, integrating cultural values into health-promoting campaigns can be viewed as a holistic approach to achieving health equity for all. For instance, Watzlawick et al. introduced the multidimensional transaction breakup of the health communication concept - the content and the relationship [17]. The content entails the intended message, and the relationship includes the dynamics between the sender and receiver. This division purposely differentiates between two arms of a health message - the actual meaning of the message and the cultural factors playing in the background. Cultural integration into health promotion programs is not de novo and specifically in KSA; the topic has been discussed and important for over a decade. Studies discussing cultural considerations and health education date back to 1990 [18]. In addition, the campaigns were not created abruptly without background evidence. A study by Al-Bannay et al. conducted in 2015 showed that a culturally tailored program in diabetic or at-risk women improved blood glucose levels, functionality, quality of life, and knowledge of diabetes and its proper management [19].

Since social media, the new era constitutes an integral part of the Saudis’ daily life and activities, and it has had a huge role in communicating health and healthcare throughout the past years and until today. Studies manifesting the role of social media and the preference for social media as a health transmitter were evident in two studies done by Bahkali et al. and El Tantawi et al. about receiving health education by women and oral health information by adolescents, respectively [20,21].

The KSA MoH was able to meet this cultural interference, in addition to social media inclusion, which was mainly through Twitter, as mentioned earlier in this paper, particularly during the COVID-19 vaccination campaigns. Through social media platforms, we not only guarantee an appealing interface between Saudis and health programs but also we are sure that it will reach the highest number possible of the public and propagate swiftly.

## Conclusions

Communicating health messages is a state-of-the-art essential to ensure the effective transmission of health information and recommendations. Effective communication is achieved by adequately delivering health messages that should be sound to the targeted populations. Ultimately, health awareness campaigns aim to improve health by changing risky behaviors and adopting healthy ones. Achieving this end through integrating the reality of individuals into health awareness campaigns received massive acceptance from the public with high impact. Saudi MoH successfully adopted culturally adapted health campaigns and witnessed significant accomplishments.
